# Recruiting Migrants for Health Research Through Social Network Sites: An Online Survey Among Chinese Migrants in Australia

**DOI:** 10.2196/resprot.3960

**Published:** 2015-04-27

**Authors:** Jie Hu, Kam Cheong Wong, Zhiqiang Wang

**Affiliations:** ^1^Center for Chronic DiseaseSchool of MedicineUniversity of QueenslandHerstonAustralia; ^2^University of Western SydneySydneyAustralia; ^3^University of SydneySydneyAustralia

**Keywords:** Chinese migrants, online survey, recruiting

## Abstract

**Background:**

Traditionally, postal surveys or face to face interviews are the main approaches for health researchers to obtain essential research data. However, with the prevalence of information technology and Internet, Web-based surveys are gaining popularity in health research.

**Objective:**

This study aims to report the process and outcomes of recruiting Chinese migrants through social network sites in Australia and to examine the sample characteristics of online recruitment by comparing the sample which was recruited by an online survey to a sample of Australian Chinese migrants collected by a postal survey.

**Methods:**

Descriptive analyses were performed to describe and compare the process and outcomes of online recruitment with postal survey questionnaires. Chi square tests and *t* tests were performed to assess the differences between the two samples for categorical and continuous variables respectively.

**Results:**

In total, 473 Chinese migrants completed the online health survey from July to October 2013. Out of 426 participants recruited through the three Chinese social network sites in Australia, over 86.6% (369/426) were recruited within six weeks. Participants of the Web-based survey were younger, with a higher education level or had resided in Australia for less time compared to those recruited via a postal survey. However, there was no significant difference in gender, marital status, and professional occupation.

**Conclusions:**

The recruitment of Chinese migrants through social network sites in our online survey was feasible. Compared to a postal survey of Chinese migrants, the online survey attracted different group of Chinese migrants who may have diverse health needs and concerns. Our findings provided insightful information for researchers who are considering employing a Web-based approach to recruit migrants and ethnic minority participants.

## Introduction

Migrant and ethnic minority groups often have poorer self-perceived health status than the general population, and recruiting participants from those groups for health research is challenging [1]. Migrants and ethnic minorities, especially those who were originally from non-English speaking countries, are likely to be underrepresented in population-based national health surveys due to language and cultural barriers and low health literacy [2-4]. With the rapid development of information technology, Internet-based surveys are gaining popularity in health research projects [5]. It is expected that such surveys will become a promising alternative approach for recruiting research participants in health research realms [6].

Conventional population-based sampling is usually costly and time consuming in producing sufficient numbers of minorities [4]. Many health studies in migrants and ethnic minorities have adopted purposeful sampling methods to recruit participants in specific premises including clinics, churches, and community centers [7,8]. In some studies, participants were recruited through a telephone directory by selecting surnames which might indicate the person’s ethnic background [9,10]. Nevertheless Smith et al. proposed that a telephone directory sampling strategy was less likely to include subjects born outside of Australia than a door-to-door population census [11].

Web-based or Internet-based data collection methods have been credited for the speed with which data can be collected, low cost, and direct data entry in comparison with traditional paper-based questionnaires [6]. The recent success of Web-based recruiting for young women in Australia shows that using the Internet in medical research presents an opportunity for innovative recruitment modalities [12]. However, a study conducted in the United States successfully recruited a targeted number of white cancer patients from wider Internet communities but failed to recruit a sufficient number of ethnic minority cancer patients from ethnic-specific Internet communities [13].

Social network sites (SNSs) include all types of online social platforms that allow participants to connect and interact within broader Internet communities [14,15]. There are an increasing number of studies using social network sites as a tool or a platform in the public health domain with only a small number describing the use of SNSs among migrants or ethnic minorities. The use of the Internet in recruiting may present broader opportunities to engage ethnic minority groups in health research in order to understand their special health needs and to reduce health inequalities. A previous study found that participants of Web-based surveys were younger and had a higher education level than those of paper-based surveys [16]. It is of interest to understand whether the data collected through the SNSs are comparable to those obtained through traditional data collection methods among migrants and ethnic minorities.

Australia is a culturally-mixed country with more than a quarter of the population born overseas [17]. There is an increasing demand for health research among this growing migrant population. This paper summarizes the outcomes of recruiting Chinese migrants through social network sites and examines the sample characteristics of online recruitment by comparing the sample which was recruited by an online survey with the sample collected by a postal survey respectively amongst the Australian Chinese migrant communities. The findings may provide some insights to researchers who are considering a Web-based approach to recruit migrants and ethnic minority participants.

## Methods

### Online Survey Procedure

Participants who self-identified as Chinese and had been living in Australia longer than 3 months were recruited through several social websites to complete an online health survey from July to October, 2013. A structured questionnaire with 57 questions regarding demographics, health related risk behaviors, health service use, antibiotic use, and mental problems was uploaded to the University of Queensland server using online survey software [18]. Participants were directed to the survey webpage by clicking a link provided in a series of ads. Before proceeding to the survey, participants were required to provide consent by stating that they understood the provided information and agreed to participate in the study. The opportunity to win 1 of ten $50 gift cards was provided as an incentive for completing the online survey. Participants who completed the questionnaire and provided their name and email address were automatically entered into the draws. The lottery draws occurred five times over the entire survey period when the total number of participants reached 80, 160, 240, 360 and at the endpoint of the survey. Two participants were chosen each time, and were announced as draw winners in the recruiting threads by displaying part of the email address of the winners. At the end of the survey, a brief summary of the survey results was emailed to each participant. This study was approved by University of Queensland School of Medicine Low Risk Ethical Review Committee (2013-SOMILRE-0074).

Our recruitment posts with detailed survey information were initially posted on 3 major Chinese social network sites, including ”Oursteps”, ”Ozyoyo”, and ”Freeoz”. In addition, those notices were also posted on other Chinese social network sites such as “Yeeyi”, “ozchinese”, QQ instant message, and Weibo. “Oursteps” and “Freeoz” had members Australia-wide while “Ozyoyo” is self-claimed to be the largest Chinese website in Queensland. The primary language of all these sites is Simplified Chinese.

### Postal Survey Procedure

The data collected in this online survey were compared with a data set of Australian Chinese respondents collected through a postal survey from November 2005 to February 2006. The process of the postal survey was reported elsewhere [19]. Briefly, an invitation letter and a bilingual (Chinese and English) survey questionnaire along with a self-addressed stamped envelope were mailed to 500 migrants assumed to be Chinese, randomly selected through a Brisbane telephone directory by identifying possible Chinese surnames. No financial incentives were provided in this study; however, a health information pamphlet was included in the mail. In total, 213 participants returned the completed questionnaire. Data were entered manually by the second author (KW).

### Data Analysis

The survey software was able to capture some information about participation such as IP addresses of each participants, as well as date and time of starting and finishing the survey. Data were exported directly from the survey software as Excel files. As all analyses were performed using Stata 13 [20], Excel files were then converted to a compatible format for analysis. Descriptive analyses were performed to describe and compare the process and outcomes of online recruitment with postal survey questionnaires. Chi square tests for categorical variables and *t* tests for continuous variables were conducted.

## Results

### Online Recruiting Outcomes

**Figure 1:**
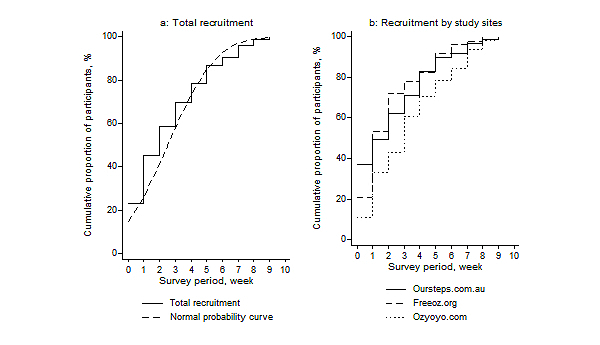
Increasing number of participants over the ten-week recruitment period (total recruitment and recruitment by three SNSs).

Of the 600 Chinese migrants who visited the survey webpage, 473 (78.8%) completed the online questionnaire. While online recruiting was set to be closed on September 30^th^, 2013, notices were not removed from the SNSs after the closing date, and we still received a few completed questionnaires, which were included in the final total number of participants. During the total survey period (July-October, 2013), the advertising posts were viewed more than 8000 times including repeated viewings and administrative viewings. By the end of September, 426 out of 473 (90%) participants were recruited through 3 Chinese social network sites “Oursteps”, “Freeoz”, and “Ozyoyo”; 35 migrants were recruited through other social network sites. There were an additional 12 completed questionnaires received between September 30^th^and October 30^th^, 2013. Table 1 provides details of the viewings and respondents through several SNSs. Figure 1 shows the increasing number of participants over the 10-week recruitment period through the 3 SNSs (total recruitment and recruitment by study sites). As we can see in Figure 1, the recruitment outcome through the 3 websites reached over 86.6% (369/426) of the total participants in 6 weeks.

We provided 10 50 Australian Dollar (AUD) gift cards presented as lucky draw winners. Each gift card was mailed to the winner who was randomly selected from the valid participants. The postage for 10 registered mails was AUD44. No additional costs were incurred, as the University of Queensland provided free access to the university’s server and the survey software.

### Identification of the Online Survey Participants

The online survey software enabled us to capture the IP address of each participant. IP addresses identified 94.1% (445/473) of the participants as living in Australia when they undertook the survey. Around 2.5% (12/473) of the participants’ IP addresses were outside Australia, and the remaining 3.4% (16/473) of the IP addresses were not detectable. Seven pairs of duplicate IP addresses were detected, which were further verified as different participants (with different personal information such as gender and age).

The selection criteria for our online survey include “self-claimed as Chinese” and “have resided in Australia for longer than 3 months”. Out of 473 total participants, 440 (93.0%) were born in Mainland China. Others were born in Taiwan, Hong Kong, Macau, and Malaysia. Among the total participants, 4 of them reported living in Australia for less than three months and another 7 did not answer the question about their length of residence in Australia.

### Sample Characteristics and Comparisons With Data From a Postal Survey

Participants of the online survey were spread over of Australia including Queensland, New South Wales, South Australia, Victoria, and West Australia. Fifty-five percent were females. The mean age of participants in the Web-based survey was 33.1 (SD 8.2) years. Table 2 shows the demographic characteristics of the 2 samples of Australian Chinese migrants collected through the online survey and the postal survey, respectively. The Web-based survey participants were substantially and significantly younger (by about 12 years) than those of the postal survey. The proportions of participants in different age groups were significantly different between the 2 samples. Of those who completed the Web-based survey, 80.2% (372/464) were under 40 years old, with only 35% of participants in the postal survey being in the same age bracket. The average length of stay in Australia of Web-based survey participants was 5.8 years (ranging from 1 month to 27 years) and was 8.3 years shorter than the postal survey participants (14.1 years, ranging from 6 months to 54 years).

Over 90% of total participants in the Web-based survey (440/473) were born in mainland China versus only 31% in the postal survey. The proportion with an undergraduate or higher degree was significantly higher in the Web-based survey group (85.6%, 405/473) than in the postal survey group (75%). A substantial proportion of the participants had a family doctor in the postal survey (73%) compared to the Web-based survey (47.1%, 223/473). However, there was no significant difference in gender, marital status, BMI, or professional occupation between the 2 samples.

**Table 1:** Recruiting details in Chinese social network sites from July to October 2013^a^.

Study websites	Start	End	Number of viewings^b^	Valid participants
Oursteps	23/07/2013	30/09/2013	2774	146
Freeoz	22/07/2013	30/09/2013	1861	135
Ozyoyo	30/07/2013	30/09/2013	2290	145
Yeeyi	04/09/2013	30/09/2013	2109	22

^a^13 participants recruited from several other websites plus 12 participants recruited after the deadline had passed were not included in the table.

^b^Including duplicated viewings

**Table 2:** Comparison of demographic characteristics between the 2 samples of Australia Chinese migrants, n (%) or mean (SD).

Characteristics		Online survey	Postal survey	*P* value
		mean (SD) or n (%)	mean (SD) or n (%)	
Total participants^a^		473	213	
**Age, years, n (%)**				
	mean (SD)	33.1 (8.2)	45.1 (14.3)	<.001
	<30	158 (34.1)	36 (18.5)	<.001
	30-39	214 (46.1)	30 (15.5)	
	40-49	71 (15.3)	62 (32.0)	
	≥50	21 (4.5)	66 (34.0)	
Female		261 (55.2)	116 (56.0)	.84
Born in Mainland China, n (%)		440 (93.2)	66 (31.0)	<.001
Undergraduate or higher degree		405 (86.2)	157 (74.8)	<.001
Professional occupation ^b^		206 (43.6)	86 (42.2)	.72
Married or in a partnership		352 (74.7)	144 (78.3)	.34
**Length of residing in Australia, years, n (%)**				
	Mean (SD)	5.8 (4.2)	14.1 (8.8)	<.001
	≥5	264 (55.8)	182 (85.5)	<.001
BMI, mean (SD)		23.2 (4.4)	22.6 (3.1)	.08
Have a family doctor, n (%)		223 (48.2)	155 (72.8)	<.001
Family doctor speaks same language, n (%)		133 (59.6)	108 (73.0)	.008

^a^The sum of participants in subgroups may not equal to the total number of participants due to missing values.

^b^Non-professionals include administrative positions, those with home duties, skilled laborers, the unemployed, pensioners, manual laborers, and the self-employed.

## Discussion

### Principal Findings

Overall, it is feasible to recruit large numbers of community-based Chinese migrants through social network sites with minimal cost. The majority of participants were recruited within 6 weeks through 3 major Chinese SNSs. Participants of the online survey were younger or had resided in Australia for a relatively shorter time than those of the postal survey. However, participants’ gender, marital status, and professional occupations were comparable.

In this online survey, we recruited over four hundred Chinese migrants. Based on a review of the postal codes of participants, we determined that the Chinese migrants resided dispersedly in 5 different states of Australia. The survey was initially designed to recruit Chinese participants through the 3 major Chinese SNSs, where the principal investigator is an active member of the SNS. We then extended our study to include other Chinese SNSs where the principal investigator is a newly registered member. As of September 30^th^, 2013, 426 participants were recruited from the three social network sites and 35 participants were recruited from other sites. As we can see in Table 1, the recruiting thread was viewed over 2000 times with only 22 valid participations in 4 weeks’ time through “Yeeyi”. Community coordinators or existing relationships in the community have been proven to be helpful in recruiting ethnic minorities [21]. Our study found similar scenarios among the Internet communities; recruiting outcomes were less satisfactory when the survey initiator was a fresh member in the Internet community.

We noted that the majority (86.6%, 369/426) of participants were recruited within 6 weeks of the survey period and the incoming numbers of respondents slowed down dramatically thereafter. Findings supported the assumption that online survey responses would be quicker compared to traditional mail surveys [5]. Our recruiting message was posted in several social websites as a thread which would be pushed down towards the bottom of the page by new threads or any existing threads which received new replies. In a SNS with a high number of active members, threads are constantly being created or replied to at a fast pace. It is therefore important to ensure the recruiting thread is attended regularly so it will not be ignored [22]. Thus we believe the first few weeks are most important in order to recruit a greater number of participants in social network sites.

We provided incentives to participants in the form of 10 lottery draws for gift cards worth AUD50 each. The marginal cost for this survey was close to AUD1.20 per participant plus organizational expenses, comparable to the cost of the postal survey (AUD0.60 postage plus organizational expenses). Since the financial incentive was relatively small and probability based, it was not likely to be a major factor in deciding to participate in the online health survey. However, by announcing the lottery winners multiple times over the survey period, we were able to attract more attention to the recruiting threads in the SNSs and expand the viewing audience.

The online survey attracted younger participants with a higher educational level than the postal survey, consistent with the existing literature that older participants with lower education might be underrepresented in Web-based surveys due to the disparity in Internet and computer access [23]. Another study comparing Web-based and paper questionnaires also found the Web-based sample to be nearly 9 years younger with a higher educational background than the paper-based sample [16]. In addition, Smith et al reported that samples based on telephone directories are very likely to exclude younger participants and participants who do not own property [11].

As regards migrant health studies, the length of stay in a host country is an important indicator of acculturation. In this study, we found that 85% of participants in the postal survey had been residing in Australia for more than 5 years, compared with about half of participants in the Web-based survey. Similarly, studies amongst US migrants found samples of longer-stay migrants using a telephone directory list [10]. Due to immigration regulations, new arrival migrants are generally young and well educated. They are likely to seek social support through the Internet in the early stage of their immigration [24]. Consequently, Web-based health research could be a promising supplementary method to reach the newly-arrived migrants.

In addition, we found the percentage of the participants having a family doctor was significantly higher in the postal survey than in the Web-based survey. Compared to Web-based survey participants, the postal survey participants were more likely to have a family doctor who could speak the same language as themselves. The findings indicate that health-related behavior may be very different due to various demographic features of the 2 samples. Researchers need to be aware of such differences when interpreting findings from different survey approaches.

Nevertheless, the online survey approach enabled us to quickly recruit Chinese migrants who were living in different states of Australia within a specific limited time and budget. The findings reported here regarding recruiting processes and outcomes are strictly applicable to these specific Chinese social network sites. More studies will be needed to articulate how to effectively recruit migrants or ethnic minorities of various populations using the Internet.

### Limitations

We also identified several limitations when we compared the data of these 2 surveys. First, these 2 health surveys adopted different strategies which may have contributed to the discrepancy in the composition of the 2 samples. The participants of postal survey included Chinese who were born in Hong Kong, Taiwan, Malaysia, Singapore, and other countries. The majority of the participants of online survey were born in Mainland China because that is where the majority of the registered members of the study websites were from. Second, the incentive of winning a gift card was offered in the online recruitment, whereas no financial incentives were provided in the postal survey. However, since the incentive is small and probability-based, we believe it is unlikely to be the major motivation of taking part in the health survey. Third, due to convenience sampling, neither sample is representative to the whole Australian Chinese community. Fourth, access to the online survey was confined to participants with adequate computer literacy to complete a questionnaire online. Our recruiting through social network sites was likely to overlook those older Chinese migrants who do not use computers and Internet. Last but not least, the postal survey was conducted 7 years prior to the Web-based survey. The demographic composition of migrants might have changed over time. Even so, however, that potential change in demographics is unlikely to account for the observed differences between 2 surveys. A recent study in the United States, using a telephone directory to obtain a sample of Chinese migrants, also showed that the sample population of a postal survey was relatively older and they were longer-stay migrants [10].

### Conclusions

Recruiting Chinese migrants through social network sites for health research is demonstrated to be feasible in our online survey. This paper provided detailed processes and outcomes for recruiting Chinese migrants through social network sites in Australia. This online recruitment method is likely to reach younger or shorter-stay Chinese migrants but may miss those older or longer-stay migrants who may not use the Internet. Researchers need to be cautious of this potential sampling bias when interpreting their results. Nevertheless, these findings could be useful for planning and conducting future research or intervention programs among migrants and ethnic minorities.
